# Kidney dysfunction due to AA amyloidosis in a morbidly obese female 

**DOI:** 10.5414/CNCS111133

**Published:** 2023-07-28

**Authors:** Hassan Izzedine, Abhishek Nimkar, Joyita Bharati, Isabelle Brocheriou, Alexis Mathian, Frederic Charlotte, Kenar D. Jhaveri, Sophie Georgin-Lavialle

**Affiliations:** 1 Department of Nephrology, Peupliers Private Hospital, Paris, France,; 2 Division of Kidney Diseases and Hypertension at Northwell Health, Donald and Barbara Zucker School of Medicine, Northwell Health, NY, USA,; 3 Department of Pathology,; 4 Department of Internal Medicine, Pitie Salpetriere Hospital,; 5 Department of Internal Medicine, Tenon Hospital, Paris, France, and; 6 Glomerular Center at Northwell, Northwell Health, NY, USA

**Keywords:** obesity, inflammation, amyloidosis, serum amyloid A

## Abstract

Kidneys are commonly involved in systemic amyloidosis. Systemic AA amyloidosis is known to be associated with states of chronic inflammation such as autoimmune conditions, chronic infections, and malignancies. Obesity is increasingly recognized to be a risk factor for low-grade, chronic inflammation. We report a 48-year-old female with morbid obesity who presented with unexplained persistent mild kidney dysfunction and low-grade proteinuria. Attempt at evaluating the cause of kidney dysfunction included performing kidney biopsy despite technical challenges. Kidney biopsy showed AA amyloidosis with predominant vascular deposition, explaining the absence of nephrotic-range proteinuria. Evaluation for secondary causes of systemic AA amyloidosis was negative. While our patient was treated with sleeve gastrectomy for morbid obesity with reasonable response, it is likely that ongoing chronic inflammation, reflected by her laboratory markers, resulted in AA amyloidosis. Treatment with anakinra, an interleukin-1 antagonist, led to improvement in the laboratory markers in the next 6 months, and her kidney function remained stable. This report highlights an important cause of kidney dysfunction in morbid obesity, an atypical presentation of AA amyloidosis, and emphasizes the value of kidney biopsy in such patients.

## Introduction 

Amyloidosis is a group of diseases characterized by extracellular deposition of insoluble fibrils leading to organ dysfunction [1]. Fibrils are β-pleated sheet structures derived from different proteinaceous sources, and each type of fibril characterizes a specific type of amyloidosis. AA amyloidosis, characterized by deposition of fibrils composed of serum amyloid A protein (SAA), an acute-phase reactant, is commonly seen in conditions associated with chronic inflammation. Common conditions known with systemic AA amyloidosis are rheumatoid arthritis, inflammatory bowel disease, chronic infections, familial Mediterranean fever, and some types of malignancies. Obesity is increasingly recognized to be a chronic inflammatory state that is associated with AA amyloidosis [2]. We report an atypical presentation of renal AA amyloidosis in a morbidly obese patient who did not have any commonly associated underlying chronic inflammatory condition on extensive evaluation. 

## Case 

A 48-year-old Maghrebian woman sought opinion for persistent kidney dysfunction for the last 5 years. Her past medical history is remarkable for a sleeve gastrectomy for morbid obesity followed by a drop in body mass index from 58.6 to 40.2 kg/m^2^ 8 years ago. Our patient had visceral type obesity with a gynoid phenotype. She took ibuprofen pills intermittently for mechanical joint pains related to obesity, on an average 1 – 2 tablets a week for the past 10 years. There was no history of recurrent chronic infections, chronic inflammatory disease, or malignancy. 

The patient was aware of her kidney dysfunction for the last 5 years with her serum creatinine values ~ 1.5 mg/dL. At the most recent visit, her serum creatinine was 1.47 mg/dL. She had low-grade proteinuria (urine protein-creatinine ratio of 0.4), microscopic hematuria (5 – 6 per high power field) and aseptic leukocyturia (6 – 8 per high power field). Her blood pressure was 110/75 mmHg. Serum protein electrophoresis and immunoelectrophoresis were normal. Immunological tests including serum anti-nuclear antibody, anti-neutrophil cytoplasmic antibody, serum anti-double stranded DNA antibody, HLA-B27, anti-saccharomyces cerevisiae antibody, and anti-cyclic citrullinated peptide antibody were negative. Serum complement (C3 and C4) levels were within normal range. Serum free light chain κ-λ ratio (0.9) was normal. Infectious work-up including hepatitis B surface antigen, hepatitis C core antibody, human immunodeficiency virus, and QuantiFERON-Gold TB were all negative. Her C-reactive protein (CRP) was 19.4 mg/L (normal range: < 10 mg/L), and serum haptoglobin was 2.24 g/L (normal range: 0.41 – 1.65 g/L). 

**Figure 1:**
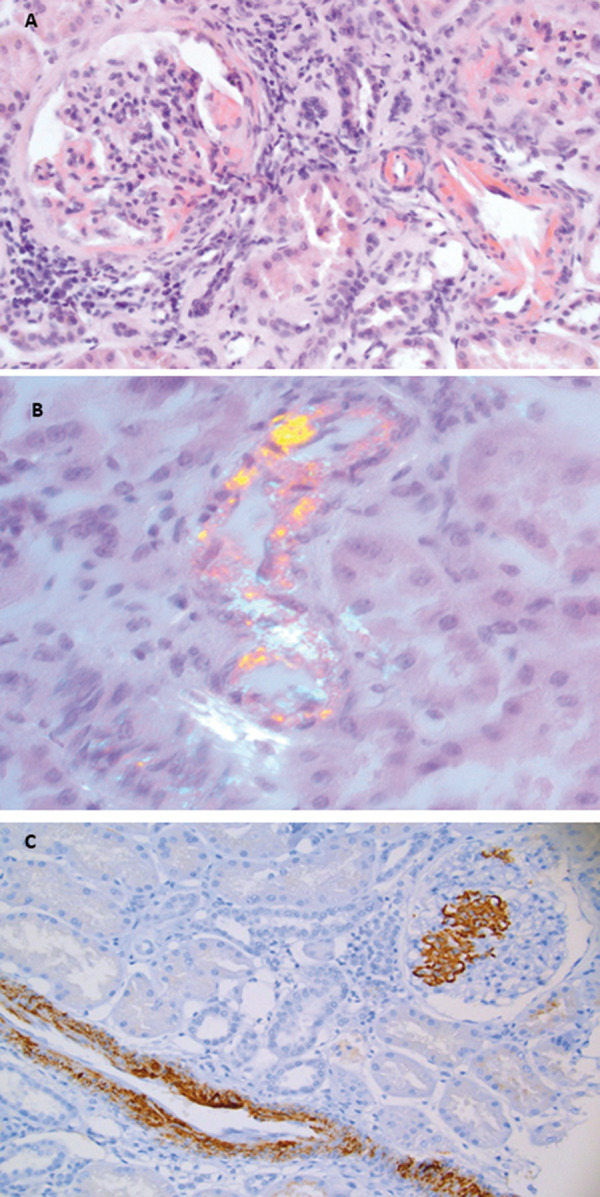
Obesity-associated vascular and mesangial AA amyloidosis. Congo red staining highlights mainly vascular amyloid deposits and scant amyloid deposition in the mesangium (A) with apple-green birefringence under polarized light (B). The amyloid stained strongly with anti-SAA antibody (C).

While the clinical picture (low-grade proteinuria, stable chronic kidney disease) raised the possibility of chronic involvement of the tubulo-interstitial compartment, likely secondary to chronic non-steroidal anti-inflammatory drug use, the background morbid obesity and inflammatory state in this young person could suggest other possibilities such as obesity-related glomerulopathy and immune-mediated glomerular diseases. A kidney biopsy was performed using the trans-jugular approach in view of anticipated technical challenges of accessing the kidneys in morbid obesity. Eight out of the total 13 glomeruli were sclerosed. The rest of the glomeruli had accentuated mesangium without any necrosis or proliferation. Interstitial fibrosis was noted to involve 10 – 15% of the cortex with a scattered mononuclear infiltrate. Discrete arteriolosclerosis was prominent. Congo red staining highlighted mainly arteriolar and arterial amyloid deposits with few deposits in the mesangium (Figure 1A), and apple-green birefringence of these deposits was noted under polarized light (Figure 1B). Direct immunofluorescence did not show significant staining. The deposits stained strongly with anti-SAA antibody (Figure 1C) but not with anti-κ and anti-λ antiserum (not shown). Further workup showed serum SAA levels of 26 mg/L (normal < 6 mg/L). Genetic study for SAA gene polymorphism was negative. Sequencing of familial Mediterranean fever gene (*MEFV*) exon 10 by Sanger sequencing did not show any pathogenic mutation. The patient was on a nutritional care program aiming at weight loss, however, her BMI had stabilized at ~ 40 kg/m^2^. The patient was started on anakinra (interleukin-1 receptor antagonist) and dapagliflozin. Within the next 6 months, there was improvement of inflammatory markers with SAA decreasing to < 10 mg/L at the 6^th^ month (Table 1). We plan to continue treatment for a total of 12 months duration. We will continue to (i) monitor SAA, CRP, kidney function test and (ii) implement nutritional care at subsequent visits. Her serum creatinine and proteinuria has been stable during the follow-up. 

## Discussion 

In this report, we describe an unusual cause of kidney dysfunction in a 48-year-old female with morbid obesity. Kidney biopsy was successfully performed despite technical challenges in the presence of morbid obesity, which showed AA amyloidosis with predominant vascular deposition and scant deposition in the mesangium. The usual secondary causes of systemic AA amyloidosis were absent while the patient had laboratory evidence of inflammation. Considering morbid obesity as the cause of chronic inflammation and AA amyloidosis, treatment with anakinra was started. In the next 6 months, her laboratory parameters improved, and kidney function remained stable. 

Obesity has been identified as an independent risk factor for end-stage kidney disease [3]. Various conditions are attributed to cause kidney disease in obese patients. Obesity-related glomerulopathy (ORG) is a specific condition in obese patients known to cause proteinuric kidney disease. ORG is characterized by focal segmental glomerulosclerosis with glomerulomegaly or glomerulomegaly alone causing proteinuria and progressive glomerulosclerosis. Presentation with an estimated glomerular filtration rate of < 30 mL/min/1.73m^2^, nephrotic syndrome or acute kidney injury are not typical of ORG and should direct evaluation towards other causes of kidney dysfunction [4]. 

AA amyloidosis is a rare disease secondary to chronic low-grade inflammatory states [5]. Obesity, especially visceral obesity, has been well established to be associated with a chronic (low-grade) inflammatory state [2, 6] as well as insulin resistance [7]. Systemic low-grade inflammation in obesity is caused by upregulation of macrophage activity resulting in secretion of the pro-inflammatory cytokines (interleukin-6 (IL-6), tumor necrosis factor (TNF)-α), and chemokines (monocyte chemotactic protein-1) by adipocytes [8]. IL-6 subsequently stimulates the hepatic production of CRP and SAA [9]. Obesity-induced chronic inflammation and its association with AA amyloidosis in humans was first described two decades ago in one of two sisters with extreme obesity due to a genetic leptin receptor deficiency [10]. In a retrospective analysis of the French national center for AA amyloidosis database, 13 patients, representing 12% of all AA amyloidosis cases, had obesity (including moderate, severe, and morbid obesity) as the cause of chronic inflammation [11]. Further, Blank et al. [12] substantiated these observations by their finding on obesity as a susceptibility factor in patients with idiopathic AA amyloidosis [12]. Kidney biopsy typically reveals a glomerular pattern in 90% and vascular pattern in 10% of patients [13]. However, the percentage of vascular AA amyloidosis might be higher if kidney biopsies were performed in cases of mild kidney dysfunction with low or no proteinuria [11]. Polymorphisms in SAA1 were shown to be associated with a higher risk of AA amyloidosis in obesity. Two single nucleotide polymorphisms at exon 3 constitute three different isotypes: SAA 1.1 (Val52–Ala57), SAA 1.3 (Ala52–Ala57), and SAA 1.5 (Ala52–Val57). Patients with a 1.1/1.1 genotype have a 3- to 7-fold increased risk for amyloidosis [14]. However, no such polymorphisms were detected in our patient. 

Treatment of AA amyloidosis, which is based on reducing the SAA levels to normal values (below 3 mg/L), is associated with prolonged survival and better renal outcome [15]. Previous studies have shown improvement of obesity-associated inflammation with aggressive weight loss through diet modification, drugs, and/or bariatric surgery [8, 16, 17]. In patients with persistent organ dysfunction even after surgical intervention, treatment with biologic agents such as anti-IL-6, anti-IL-1 or TNF-α blockade can be attempted to decrease SAA levels. Our patients was treated in this line and was noted to show improvement. 

## Conclusion 

AA amyloidosis is an increasingly recognized cause of nephropathy in morbidly obese individuals that can present atypically as mild kidney dysfunction and low-grade proteinuria. Predominant vascular AA amyloid deposition is not uncommon in the setting of morbid obesity. These findings highlight the importance of kidney biopsy despite associated technical difficulty in such individuals. Aggressive weight reduction is crucial for favorable outcome. Treatment with biologic anti-inflammatory agents should be considered in cases with poor response to weight reduction strategies. 

## Funding 

No funding reported. 

## Conflict of interest 

KDJ reports consultancy agreements with Secretome, George Clinicals, PMV pharmaceuticals, GSK, and Calliditas. KDJ reports honoraria from the American Society of Nephrology and UpToDate.com; reports serving on the editorial boards of American Journal of Kidney Diseases, CJASN, Clinical Kidney Journal, Journal of Onconephrology, Kidney International, and Nephrology Dialysis Transplantation; reports serving as Editor-in-Chief of ASN Kidney News and section editor for onconephrology for Nephrology Dialysis Transplantation. 

**Table 1.** Trend of inflammatory markers with treatment.

Parameter	Initial presentation	At 3 months	At 6 months
C-reactive protein (mg/L)	19.4	23.2	7.3
Serum amyloid A protein (mg/L)	26	26	9
Interleukin-6 (IU/L)	49		3
Interleukin-18 (IU/L)	270		254
